# Diagnostic Accuracy of SARS-CoV-2 Antigen Detection Test in Children: A Real-Life Study

**DOI:** 10.3389/fped.2021.647274

**Published:** 2021-07-15

**Authors:** Camille Jung, Corinne Levy, Emmanuelle Varon, Sandra Biscardi, Christophe Batard, Alain Wollner, Patrice Deberdt, Aurélie Sellam, Isabelle Hau, Robert Cohen

**Affiliations:** ^1^Clinical Research Center, CHI Creteil, Créteil, France; ^2^Association Clinique et Thérapeutique Infantile du Val de Marne, Créteil, France; ^3^Paris Est University, IMRB-GRC GEMINI, Créteil, France; ^4^Association Française de Pédiatrie Ambulatoire, Orleans, France; ^5^Laboratory of Medical Biology, CHI Creteil, Creteil, France; ^6^Department of Pediatrics, CHI Creteil, Creteil, France

**Keywords:** children, COVID-19, real-life performance, rapid antigen test, SARS-CoV-2

## Abstract

Naso-pharyngeal RT-PCR is the gold standard for the diagnosis of COVID-19, but there is a need for rapid and reliable tests. Some validation studies have used frozen aliquots mainly from adults. The aim of this real-life study was to test the performance of a SARS-CoV-2 rapid antigen test (SC2-RAT) in children. Symptomatic patients aged 0 to 17 years were recruited in the emergency department of the University Hospital of Creteil and in primary care pediatric practices from October 10, 2020 for 7 weeks. Each enrolled child had a SARS-CoV-2 RT-PCR test and a SC2-RAT from two distinct nasopharyngeal swabs. Among the 308 patients (mean [SD] age 4.9 [5.3] years), fever was the main symptom (73.4%), with no difference between COVID-19–negative and –positive groups. The prevalence of COVID-19 was 10.7% (95% CI 7.5–14.7). On the whole cohort, the sensitivity and specificity of the SC2-RAT compared to RT-PCR was 87.9% (95% CI 71.8–96.6) and 98.5% (95% CI 96.3–99.6). Considering samples with cycle threshold >25, the sensibility was lower: 63.6% (95% CI 30.8–89.1) and the specificity 99.6% (95% CI 98.0–100.0). The mean delay to obtain an SC2-RAT result was <15 min but was 3.2 h (SD 5.5) for an RT-PCR result. Contact with a COVID-19–positive person was more frequent for COVID-19–positive than –negative patients (*n* = 21, 61.6%, vs. *n* = 64, 24.6%; *p* < 0.01). In real life, SC2-RAT seems reliable for symptomatic children, allowing to detect contagious children.

## Introduction

Since the beginning of the COVID-19 pandemic in China in early 2020, children seemed to have less severe illness than adults: fewer deaths, fewer stays in intensive care units, fewer hospitalizations (1, 2). Weeks or months later, children also seemed to be less often infected than adults and to play a minor role in the pandemic dynamic. However, although the prevalence of SARS-CoV-2 infection in children is lower than in adults, the curve for children follows the adult prevalence, and contaminations could also occur from children to other children and adults (3). Given the broad spectrum of SARS-CoV-2 manifestations in children, which are often similar to other highly prevalent viral infections in childhood, detecting SARS-CoV-2–infected children efficiently and quickly is crucial (particularly before hospitalization) in school and daycare centers but also in the family (4, 5).

Naso-pharyngeal Reverse Transcription-PCR (RT-PCR) has been the gold standard for the diagnosis of COVID-19, at least during the 1st days of the disease, when the sensitivity and specificity of this type of test is almost optimal. However, RT-PCR has several drawbacks: in addition to the inconvenience of sampling, other disadvantages are the need for equipment (thermocycler, biologist validation) and the cost and delay in obtaining results (6, 7). The delays (especially during COVID-19 waves) in RT-PCR tests being performed and results reported are sometimes incompatible with relevant decisions (4, 8). For these reasons, SARS-CoV-2 rapid antigenic tests (SC2-RATs), which share inconvenience of sampling but can be interpreted by any health professional, have been developed and have been available for several months (9). Numerous studies have attempted to assess the performance of SC2-RATs (7, 10, 11). The specificity of SC2-RATs has been good in all studies, but results are inconsistent for sensitivity, with very poor sensitivity for some (11). Despite the non-optimal sensitivity, SC2-RATs could be useful because they are easy to perform and the results are available quickly, which could be crucial for control of the epidemic (12). These studies had several drawbacks, but in our opinion, the most important is that most of these used PCR aliquots obtained several days or months before the study and the samples were often frozen, resulting in sample dilution. In addition, to our knowledge, no study has specifically tested pediatric samples. Thus, the real-world performance of these assays is uncertain, so their validation is of high priority (13).

The aim of this study was to determine the performance and accuracy of one RAT BIOSYNEX COVID-19 Ag BSS in clinical practice.

## Materials and Methods

### Study Population and Design

In this prospective study, children and youth were recruited in the emergency department of the University Intercommunal Créteil Hospital (CHI Créteil) and in three primary care pediatric practices in the Paris area from October 10 to November 27, 2020, corresponding to the second COVID-19 wave in France (14). All symptomatic children under 18 years old who required a COVID-19 RT-PCR test were eligible. The clinical criteria for performing a COVID-19 RT-PCR test in symptomatic children were previously defined by the Pediatric Infectious Pathology Group and the French Pediatric Society (15):

- Children > 6 years old presenting cough and/or fever and/or digestive disorders unless another infectious disease was diagnosed with certainty (e.g., scarlet fever, bacterial angina, enterovirus, urinary tract infection, chickenpox)
- Children <6 years old with symptoms supporting an infection such as fever, fatigue, dyspnea, diarrhea, etc. and needing hospitalization, additional examination (blood test, X-ray, etc.) or lasting more than 3 days.
- Children <6 years old who had a proven contact with a COVID-19–positive person or who were in contact at home with people considered at risk for SARS-CoV-2 infection.
- Febrile children under 3 months old.

After agreement of the parent accompanying the child, two naso-pharyngeal swabs were taken: one for a COVID-19 RT-PCR test and one for the SC2-RAT. Also, clinical data were entered into an electronic case report form: age, sex, current symptoms, duration of symptoms and history of contact with a COVID-19–positive person. When possible, the time to availability of the RT-PCR result was also collected.

### SARS-CoV-2 Antigen Detection Test

The SC2-RAT used was the BIOSYNEX COVID-19 Ag BSS (16). This test detects the presence of SARS-CoV-2 nucleoprotein in a naso-pharyngeal sample with specific monoclonal antibodies. Briefly, after sampling, the naso-pharyngeal swab is immerged in 0.3 mL (10 drops) extraction buffer for 1 min. Then, 100 μL (four drops) is dropped into the well of the test device. The test result is read in the device window after 15 min maximum: two lines (C and T) indicate a positive result; one line (C) indicates a negative result. This test has previously obtained CE marking for health, safety, and environmental protection standards.

### SARS-CoV-2 RT-PCR Methods

For patients recruited in CHI Créteil disposable sterile swabs (Zhejiang Gongdong Medical Technology, China), and Vacuette Virus Transport Tubes (Greiner Bio-One, Austria) were used to collect the naso-pharyngeal samples. The COVID-19 RT-PCR test used was the Allplex 2019-nCoV kit (Seegene, South Korea) which targets two SARS-CoV-2–specific genes (RdRP and N gene) and one Sarbecovirus specific gene from the viral envelope (17).

The pediatric private practices used the Flocked Sampling Swab (MS-96000, Miraclean Technology, Shenzhen, China) to take nasopharyngeal swabs. The swabs were immerged in 4 ml of sample collection buffer from the VitaPCRTM SARS-CoV-2 Assay kit (Trentron Biomedical Ltd, Taïwan). RT-PCR was launched immediately after sampling on the VitaPCR device (Credo Diagnostics Biomedical, Singapore) available in the doctor's office. This method amplifies two genes: one SARS CoV-2–specific gene (N gene) and one universal SARS-like gene.

In all cases, the minimum cycle threshold (Ct) value for these genes was retained for statistical analysis. RT-PCR results were considered positive if one SARS CoV-2–specific gene could be amplified with fewer than 40 cycles. In the absence of SARS-Cov2 gene amplification, the Ct value was 0 and the RT-PCR result was negative.

In case of positive SC2-RAT and negative COVID-19 RT-PCR performed on the VitaPCR device, the collected sample was controlled using the Allplex 2019-nCoV kit in the CHI Creteil microbiology lab. In case of negative SC2-RAT and positive RT-PCR, no additional controls were performed, and the result of SC2-RAT was considered as false negative.

### Ethics Consideration

The study was approved by an ethics committee (Paris Ile de France 3). Expressed consent of at least one parent was required before inclusion. The study was registered at ClinicalTrials.gov (NCT04583189).

### Statistics

Prevalence was expressed as a percentage (95% confidence interval [CI]). Sensitivity and specificity, positive and negative predictive values and positive and negative likelihood ratio with 95% CIs were calculated to compare the performance of the SC2-RAT to COVID-19 RT-PCR. Patient characteristics are described with number (%), mean (SD) or median (interquartile range [IQR]). Student *t* test, Mann-Whitney chi-square test or Fisher exact test was used for comparison, as appropriate. *P* < 0.05 was considered statistically significant. STATA 16.1/SE (StataCorp, College Station, TX, USA) was used for statistical analysis.

## Results

### Diagnostic Testing

The prevalence of COVID-19 in the cohort of 308 children was 10.7% (95% CI 7.5–14.7). As compared with COVID-19 RT-PCR, the sensitivity and specificity of the SC2-RAT was 87.9% (95% CI 71.8–96.6) and 98.5% (95% CI 96.3–99.6) (Table 1). Considering the samples with Ct value >25 (*n* = 11 patients), the sensibility was lower: 63.6% (95% CI 30.8–89.1) and the specificity 99.6% (95% CI 98.0–100.0).

**Table 1:** Performance of the BIOSYNEX COVID-19 Ag BSS compared to SARS-CoV-2 RT-PCR.

	**Value**	**95% CI**	
Prevalence	10.9%	7.6%	14.9%
Sensitivity	87.9%	71.8%	96.6%
Specificity	98.5%	96.3%	99.6%
Positive likehood ratio	59.54	22.32	158.79
Negative likehood ratio	0.12	0.05	0.31
Positive predictive value	87.9%	71.8%	96.6%
Negative predictive value	98.5%	96.3%	99.6%

*95% CI, 95% confidence interval*.

**Figure 1:**
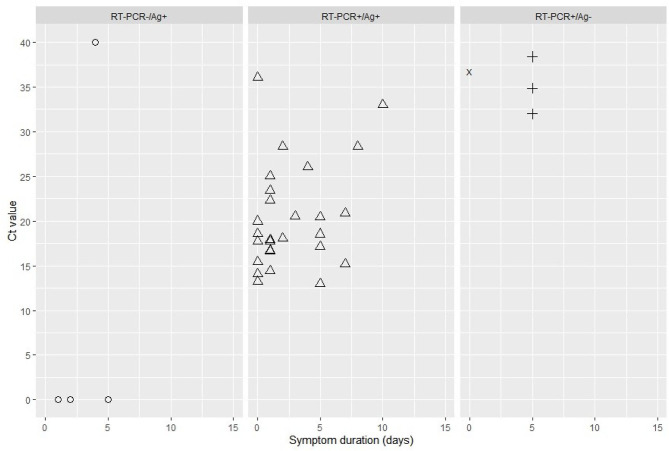
Cycle threshold (Ct) value by symptom duration (in days) for diagnostic results: RT-PCR–/SC2-RAT+ (false positive); RT-PCR+/SC2-RAT+; RT-PCR+/SC2-RAT– (false negative). The cross represents an unknown duration of symptoms. SC2-RAT, BIOSYNEX COVID-19 Ag BSS test.

Overall, 29 patients had concordant SC2-RAT and RT-PCR results and eight had discordant results (Table 2). Four patients had a negative SC2-RAT result but a positive RT-PCR result. In these cases, the median (IQR) Ct value was significantly higher (35.8 [4.1] vs. 18.5 [6.8], *p* = 0.0004) and three patients had symptoms for 5 days. For the fourth patient, these data were missing (Figure 1). Finally, four patients had a positive SC2-RAT result and a negative RT-PCR result, including one patient with an RT-PCR Ct value of 40 that was validated as negative. These four negative RT-PCR tests were controlled twice.

**Table 2:** Distribution of SARS-CoV-2 RT-PCR and BIOSYNEX COVID-19 Ag BSS (SC2-RAT) results.

	**SC2-RAT**	
**RT-PCR**	**Positive**	**Negative**
Positive	*N* = 29	*N* = 4
Negative	*N* = 4	*N* = 271

In the Ct range of 13–32 cycles, all SC2-RAT results agreed with RT-PCR results, leading to a sensitivity and specificity of 100%.

The delay to obtain an SC2-RAT result was <15 min but the mean delay was 3.2 (5.5) h for an RT-PCR result.

### Clinical Symptoms

The clinical symptoms of the COVID-19 and non-COVID-19 groups were similar (Table 3). However, contact with a person who was COVID-19–positive was more frequent for COVID-19–positive than –negative children (*n* = 21, 61.6%, vs. *n* = 64, 24.6%; *p* < 0.01).

**Table 3:** Pediatric patient characteristics according to COVID-19 status.

	**Entire cohort *n* = 308**	**COVID-19–negative^a^*****n*** **= 275**	**COVID-19–positive^b^** ***n*** **= 33**	***P*****^c^**
Age, year, mean (SD)	4.9 (5.3)	4.9 (5.2)	5.8 (6.5)	0.35
Sex, female, *n* (%)	134 (43.5)	119 (43.3)	15 (45.5)	0.81
Symptoms, *n* (%)				
Fever	226 (73.4)	199 (72.4)	27 (81.8)	0.25
Fatigue	70 (22.7)	61 (22.2)	9 (27.3)	0.50
Nasal symptoms	166 (53.9)	149 (54.2)	17 (51.5)	0.77
Pharyngitis	78 (25.3)	70 (25.5)	8 (24.2)	0.88
Cough	32 (10.3)	28 (10.2)	4 (12.2)	0.73
Otitis	8 (2.9)	8 (2.9)	0 (0.0)	1.0 ^d^
Tachypnea or dyspnea	74 (24.3)	72 (26.2)	2 (6.1)	0.01
Diarrhea	57 (18.5)	51 (18.5)	6 (18.2)	0.96
Vomiting	61 (19.8)	54 (19.6)	7 (21.2)	0.83
Decreased food intake	74 (24)	64 (23.3)	10 (30.3)	0.37
Duration of symptoms, days, mean (SD)	3.5 (5.0)	3.6 (5.3)	2.8 (2.7)	0.42
Duration of fever, days, mean (SD)	2.3 (4.3)	2.4 (4.6)	1.9 (2.6)	0.58
Contact with COVID-19–positive person, *n* (%)	85 (28.9)^e^	64 (24.6)	21 (61.6)	**<0.001**
Hospitalization after consultation, *n* (%)	65 (21.5)	59 (22.0)	6 (18.2)	0.7

a *RT-PCR–negative*;

b *RT-PCR positive whatever the result of BIOSYNEX COVID-19 AgBSS*;

c *Chi-square test*;

d *Fisher exact test*;

e *17 parents did not know. p < 0.05 are in bold*.

## Discussion

To the best of our knowledge, this is the first study assessing the performance of an SC2-RAT in real life for symptomatic children. The results are encouraging, with good sensitivity (87.9%), excellent specificity (98.5%) and 97.4% overall agreement (300/308 patients). Furthermore, in the Ct range of 13–32 cycles, all SC2-RAT results agreed with RT-PCR results, for a sensitivity and specificity of 100% in this range.

These antigenic tests seem reliable for the diagnosis of SARS-CoV-2 infection in children with symptoms; the test was false eight times (2.6% of cases) but only for cases with Ct ≥ 32 which can be considered as having a low viral load (four patients). Higher viral loads associated with better antigen detection rates was previously demonstrated (10, 18–20). Thus, in our study the sensitivity of the SC2-RAT appears good, and even excellent when focused on high viral loads. This SC2-RAT test likely detects the most contagious patients. In case of positive SC2-RAT and negative RT-PCR, a third assay to confirm the COVID-19 positivity should be used.

In their validation study of the SD-Biosensor antigen test for SARS-CoV-2, Cerutti et al. cultured discordant samples (Ag–/RT-PCR+ with Ct >30). None of these samples grew, which suggested that these patients were no longer infectious (18). The importance of taking into account viral load to interpret RT-PCR results has been underlined by other authors, and patients who have been symptomatic for a few days and retain a Ct value ≥34 are considered no longer contagious (21).

Moreover, with the excellent specificity of these tests, a control of positive tests by RT-PCR is unnecessary. Thus, the RAT saves time in management of both the infection diagnosis and the implementation of isolation and contact tracing measures for hospitalized patients and in the community. Use of the RAT allows for detecting SARS-CoV-2 infections in symptomatic children quickly (<20 min), by any healthcare professional, and reliably, especially if the symptoms started in the last 4 days.

The accuracy was better for the RAT used in our study than previously found (11). This finding may be related to the quality of the monoclonal antibodies directed against SARS-CoV-2 nucleoprotein and/or the design of the device itself for the test but also the technique of validation. In our study, the samples were obtained under real-life conditions in that two swabs were collected at the same time. This allowed us to perform the SC2-RAT test after direct antigen extraction from the swab sample according to the manufacturer recommendations. Differently, previous validation studies used frozen aliquots of samples in transport medium, obviously resulting in sample dilution (22). In addition, the investigators in this study have been trained and were experienced. Conducting diagnostic tests by less well-trained people could increase the number of false positive tests.

The use of RATs is particularly interesting in times of both the COVID-19 epidemic and winter viruses such as respiratory syncytial virus (RSV) and influenza. As previously described (23), the clinical characteristics of the COVID-19–positive patients in this cohort were not specific (24). Only the notion of contagion with a close relative who was COVID-19–positive and the spread of the epidemic should lead to suspecting SARS-CoV-2 infection (25). In this context, patients with suspected COVID-19 must receive a rapid diagnosis by a primary care physician so that they can be isolated and their entourage tested.

Public health measures to reduce the spread of SARS-CoV-2 infection such as social distancing and mandatory face masks have also had an impact on the transmission of viruses such as RSV or influenza (26). A decrease in bronchiolitis cases was previously reported in 2020 (27, 28), but substantial RSV circulation during the summer season was observed in Australia, which is unusual (29). In this context of changing seasonality and viral transmission, it is even more important to have a rapid, simple, reliable and cheap test. The development of tests for the diagnosis of RSV, influenza and COVID-19 from single sample will be useful (30).

### Strength and Limitations

The strength of this study is showing the accuracy of an RAT in real life, under conditions of use as recommended by the manufacturer (16). Additionally, this is the first report focusing on children. The main limitation of our study is the relative low prevalence (about 10%) of SARS-CoV2 infection in our cohort, much lower than in other validation studies that used previously collected samples. Moreover, additional data including more positive samples with higher Ct value would allow to validate these first results. Actually, if SC2-RAT is negative, the sample should be repeated with highly sensitivity assay if clinically indicated.

## Conclusion

For an effective COVID-19 pandemic strategy, we need tests that can diagnose most infections while patients are still infectious. Rapid lateral-flow antigen tests allow for identifying children who are currently transmitting the virus. They allow for a quick diagnosis in symptomatic outpatients and firm implementation of isolation measures and early detection of infected contacts. They can be used in symptomatic children.

## Data Availability Statement

The datasets generated for this article are not readily available because Anonymized raw data could be available for the ediatorial board in EU but cannot be made public because the legal guardian has not agreed to it. Requests to access the datasets should be directed to camille.jung@chicreteil.fr.

## Ethics Statement

The studies involving human participants were reviewed and approved by Comité de Protection des Personnes; Paris Ile de France 3. Written informed consent to participate in this study was provided by the participants' legal guardian.

## Author Contributions

RC, CL, IH, EV, and CJ designed the research, collected the data, performed the statistical analyses, interpreted the data, and wrote the manuscript. SB, CB, AW, PD, AS, RC, and IH recruited the patients and collected the data. All authors critically reviewed and approved the manuscript.

## Conflict of Interest

The authors declare that the research was conducted in the absence of any commercial or financial relationships that could be construed as a potential conflict of interest.

## Abbreviations

SC2-RAT: SARS-CoV-2 Rapid Antigen Test
RAT: Rapid Antigen Test
RT-PCR: Naso-pharyngeal Reverse Transcription-PCR
RSV: respiratory syncytial virus.
